# Structure–function characterization of an aldo–keto reductase involved in detoxification of the mycotoxin, deoxynivalenol

**DOI:** 10.1038/s41598-022-19040-8

**Published:** 2022-08-30

**Authors:** Nadine Abraham, Kurt L. Schroeter, Yan Zhu, Jonathan Chan, Natasha Evans, Matthew S. Kimber, Jason Carere, Ting Zhou, Stephen Y. K. Seah

**Affiliations:** 1 Department of Molecular and Cellular Biology, University of Guelph, Guelph, Canada; 2 Guelph Research and Development Centre, Agriculture and Agri-Food Canada, Guelph, ON Canada

**Keywords:** Oxidoreductases, Environmental biotechnology

## Abstract

Deoxynivalenol (DON) is a mycotoxin, produced by filamentous fungi such as *Fusarium graminearum*, that causes significant yield losses of cereal grain crops worldwide. One of the most promising methods to detoxify this mycotoxin involves its enzymatic epimerization to 3-*epi*-DON. DepB plays a critical role in this process by reducing 3-keto-DON, an intermediate in the epimerization process, to 3-*epi*-DON. DepB_Rleg_ from *Rhizobium leguminosarum* is a member of the new aldo–keto reductase family, AKR18, and it has the unusual ability to utilize both NADH and NADPH as coenzymes, albeit with a 40-fold higher catalytic efficiency with NADPH compared to NADH. Structural analysis of DepB_Rleg_ revealed the putative roles of Lys-217, Arg-290, and Gln-294 in NADPH specificity. Replacement of these residues by site-specific mutagenesis to negatively charged amino acids compromised NADPH binding with minimal effects on NADH binding. The substrate-binding site of DepB_Rleg_ is larger than its closest structural homolog, AKR6A2, likely contributing to its ability to utilize a wide range of aldehydes and ketones, including the mycotoxin, patulin, as substrates. The structure of DepB_Rleg_ also suggests that 3-keto-DON can adopt two binding modes to facilitate 4-*pro*-R hydride transfer to either the *re*- or *si*-face of the C3 ketone providing a possible explanation for the enzyme’s ability to convert 3-keto-DON to 3-*epi*-DON and DON in diastereomeric ratios of 67.2% and 32.8% respectively.

## Introduction

Mycotoxins are toxic secondary metabolites produced by filamentous fungi that pose a serious health safety risk for humans and livestock^1^. Deoxynivalenol (DON), belongs to a class of *Fusarium* mycotoxins termed trichothecenes which share a tetracyclic sesquiterpene carbon skeleton^2^. Commercial cereal grain crops infected by toxigenic *Fusarium* species show diminished quality, and yield, and often contain high levels of DON. The FDA estimates crop losses due to mycotoxins in the US alone, to be around $932 million annually, fueling the need for more effective management strategies^3^.

**Figure 1:**
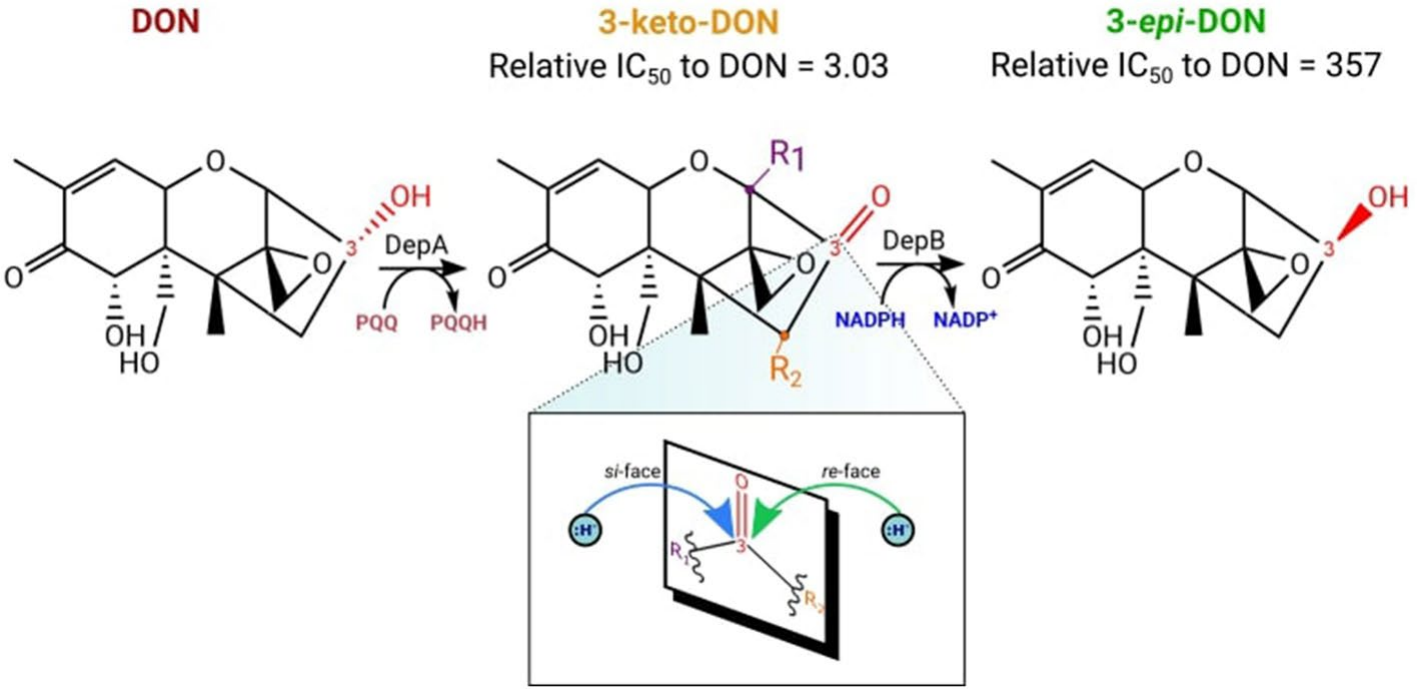
The Dep pathway. The C3 OH group of DON (shown in red) is stereochemically inverted to produce the diastereomer, 3-*epi*-DON. In the expanded image of this stereogenic center, R_1_ and R_2_ represent the priority groups attached to the *sp*^2^ hybridized C3. Hydride attack on the *re*-face at the prochiral C3 center generates 3-*epi*-DON.

DON is a potent protein synthesis inhibitor that blocks the A-site of ribosomes, hindering aminoacyl tRNA binding and preventing peptidyl transferase activity^4^. The ribosome interaction is mediated through hydrogen bond contacts from the C12, 13 epoxide ring, C3 hydroxyl, and van der Waal contacts between the C9, C10 double bond^5^. Together, these substituent groups contribute to DON’s overall toxicity. Certain bacteria, such as *Devosia mutans* 17-2-E-8 enzymatically detoxify DON by stereochemically inverting the C3 OH from the (S)-configuration to the (R)*-*configuration producing the diastereomer, 3-*epi*-DON^6^. This impairs binding to the ribosome and overall, reduces its toxicity; as evidenced by MTT bioassays, 3-*epi*-DON possesses an IC_50_ 357 times higher than DON^7^. This process, termed ‘**D**ON **ep**imerization’ (Dep) occurs in two stages^8^. First DepA, a PQQ-dependent alcohol dehydrogenase oxidizes the C3 OH group of DON to form 3-keto-DON^9^. A second enzyme, DepB, then reduces 3-keto-DON at the *re*-face of this stereogenic center to produce 3-*epi*-DON^8,10^ (Fig. 1). The intermediate, 3-keto-DON is only moderately less toxic than DON with an IC_50_ 3-times higher than DON^7^. Therefore, the critical step of DON detoxification is catalyzed by DepB.

DepB belongs to the aldo–keto reductase (AKR) family of enzymes. These ubiquitous oxidoreductases occur in all domains of life, and they catalyze the reduction of diverse carbonyl substrates. They play vital roles in steroid hormone metabolism^11^, oxidative stress responses^12,13^, xenobiotic detoxification^14^, and are implicated in certain cancers^15^ and metabolic disorders such as diabetes^16,17^. Most AKRs are monomeric proteins with molecular weights ranging from 34 to 37 kDa and predominantly utilize NADP(H) as their coenzyme.

Here we present the crystal structure of DepB from *Rhizobium leguminosarum* (herein designated as DepB_Rleg_). We determined that DepB_Rleg_ belongs to a recently discovered family of AKRs and although it can use either NADH or NADPH as a coenzyme, the structural basis for its strong preference for NADPH is unknown. Although the stereochemistry of hydride transfer is well established for AKRs, the stereoselectivity of the reduction process, which is key to detoxification, is a less explored avenue. We, therefore, probed the determinants of DepB_Rleg_’s unusual coenzyme specificity and propose features of the substrate-binding site which dictate the stereochemical outcome of 3-keto-DON reduction through molecular modeling studies. In addition to its role in the Dep pathway, DepB_Rleg_ is also active towards a wide range of aldehyde and ketone substrates including the mycotoxin patulin, making it a versatile biocatalyst for the decontamination of various xenobiotics.

## Results

### SSN cluster analysis of DepB_Rleg_ with AKRs

AKRs are traditionally classified into families based on sequence identity and phylogenetic analysis. The nomenclature system consists of the root symbol AKR, followed by an arabic numeral designating the family and an alphabet representing the subfamily^18^. The number of AKRs in the protein database continues to expand and to date 18 families have been identified. Protein sequences in each family share at least 40% sequence identity while sub-families share 60% sequence identity^19^. We used a protein sequence similarity network (SSN)^20^ as an alternate, less computationally demanding method to determine sequence relationships of DepB_Rleg_ with other AKR family members. For ease of computation, sequences were iteratively clustered to produce a 40% representative node network and filtered based on sequence length. The total number of sequences following the reduction of the initial dataset is 878. In this network, nodes represent protein sequences sharing 40% or more sequence identity and edges represent the pairwise alignments between protein sequences. Edges were drawn between nodes if they exceeded the prescribed stringency threshold or BLAST E-value of *e*^−57^.

**Figure 2:**
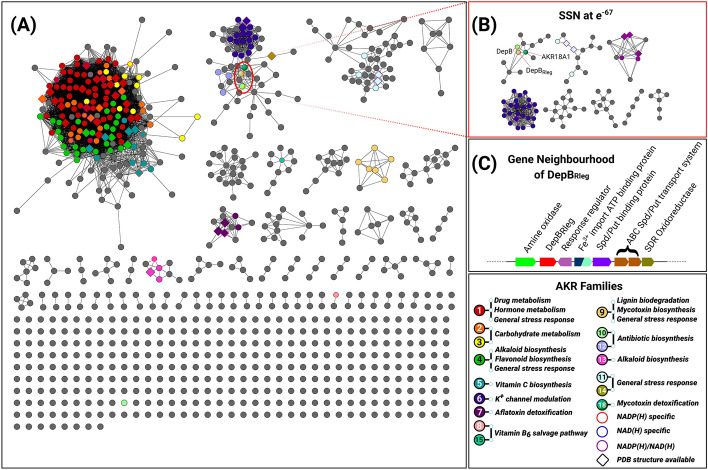
A 40% representative sequence similarity network (SSN) at a threshold of *e*^−57^. (**A**) Nodes within each cluster contain a representative protein sequence of a collection of sequences that share 40% or more sequence identity. Clusters have been annotated and color-coded based on curated protein sequences from the AKR Database^21^. Grey nodes represent AKRs that have no functional annotation. Metabolic pathways associated with the function of AKR enzymes have been color-coded to cross-reference with each cluster and numerically labeled to designate each AKR family number. The nodes of AKRs possessing biochemical data on coenzyme specificity and crystal structures have also been accordingly labeled as outlined in the legend. (**B**) SSN generated at a threshold of *e*^−67^ depicting complete segregation of the AKR18 family from AKR6, AKR12 and AKR14 members. (**C**) Genome Neighborhood Diagram of DepB_Rleg._ The GND was generated using the EFI-GNT server which depicts the coding sequence region of *depB*_*Rleg*_ along with putative functions of upstream and downstream genes.

Previous phylogenetic analysis showed that AKRs can be divided into two large groups with AKR1-AKR5 forming one branch of the phylogenetic tree and the other AKRs forming a separate branch^21^. This is generally reflected in the SSN topology where AKR1 to AKR5 family members cluster together (Fig. 2A). At this stringency threshold, there was good delineation of AKR7-11 and AKR 13-15 into distinct, isofunctional clusters. DepB_Rleg_ and its homologs from *D. mutans* 17-2-E-8^10^ and AKR18A1 from *Sphingomonas* S3-4^22^ clustered with AKR6, AKR12, and AKR14 families. At higher stringency thresholds (*e*^−67^), DepB_Rleg_ and its closest homologs resolve into a single isofunctional cluster (Fig. 2B). Curiously, in vitro assays previously revealed that AKR18A1 reduces 3-keto-DON to DON rather than 3-*epi*-DON in the presence of NADH^22^. The enzyme also reduces the C7 ketone group of the estrogenic mycotoxin, zearalenone (ZEN) to produce α-zearalenol (α-ZOL) and β-zearalenol (β-ZOL). Aside from this, AKR18A1’s substrate specificity towards other endogenous aldehydes and ketones has not been examined, nor has its crystal structure been solved.

Besides AKR18A1, the next closest homologs of DepB_Rleg_ identified from this SSN analysis were AKR12 family members based on the first stringency cut-off threshold (*e*^−57^). As per AKR classification rules, DepB_Rleg_ is not an AKR12 family member as it shares less than 40% sequence identity with enzymes from this family. In addition, these AKR12 members are involved in the biosynthetic pathways of polyketide macrolide antibiotics such as tylosin, erythromycin, and avermectin^23–25^. A genome neighborhood diagram (GND) of DepB_Rleg_ revealed the presence of a putative monoamine oxidase (MAO) upstream of the DepB_Rleg_ gene along with several putative spermidine/putrescine transporter genes downstream of DepB_Rleg_ (Fig. 2C). The neighboring genes reported for the *depB* homolog in *D. mutans* 17-2-E-8 differ from *depB*_*Rleg*_ but are also not polyketide synthesis genes^10^.

### Substrate specificity profile of DepB_Rleg_

Recombinant N-terminal His-tagged DepB_Rleg_ was purified to homogeneity by Ni-NTA chromatography. A band corresponding to a molecular weight of approximately 39 kDa was observed on a 10% SDS-PAGE gel (See Supplementary Fig. S1). The substrate specificity of the purified enzyme towards 3-keto-DON, aliphatic and aromatic carbonyl compounds, as well as nicotinamide cofactors were assessed by steady-state kinetics (Table 1). DepB_Rleg_ possesses the highest specificity constant (*k*_*cat*_*/K*_*m*_) with the diketone 9,10-phenanthrenequinone (9,10-PQ) on the order of 27 times higher relative to 3-keto-DON and 35 times higher relative to the smaller diketone, isatin. DepB_Rleg_ is also active towards endogenous toxic aldehydes derived from oxidative stress responses such as lipid peroxidation^26,27^. These aldehydes include acrolein, methylglyoxal, 4-oxo-2-nonenal, 4-hydroxy-2-nonenal, hexanal, and butanal. Among the aldehydes, DepB_Rleg_ displayed high specificity constants for the smaller unsaturated aldehydes such as acrolein and methylglyoxal, although the *K*_*m*_ for methylglyoxal was too high to be determined reliably. Among the αβ unsaturated aliphatic aldehydes, 60-fold higher specificity constant was observed for 4-oxo-2-nonenal compared to 4-hydroxy-2-nonenal. The apparent *K*_*m*_ for NADPH is about 100-fold lower than NADH (15.2 ± 1.41 µM vs. 1560 ± 399 µM) when evaluated using DL-glyceraldehyde as a substrate at a fixed concentration of 10 mM, while the apparent *k*_*cat*_ with NADPH was only 13-fold higher than with NADH (0.337 ± 0.00757 s^−1^ vs. 0.0242 ± 0.00344 s^−1^). DepB_Rleg_ was also determined to reduce the mycotoxin patulin but not citrinin. Both these mycotoxins are produced by *Penicillium expansum* which is the causative agent of blue mold rot in apples^28^. LC–MS/MS analysis indicated that patulin was indeed transformed to E-ascladiol, a by-product previously reported to be significantly less cytotoxic compared to patulin^29,30^. The mechanistic basis for this transformation has been proposed to involve the spontaneous opening of the hemiacetal ring of patulin followed by the reduction of the aldehyde to the alcohol^31^ (See Fig. S2 for LC–MS/MS results).

**Table 1:**
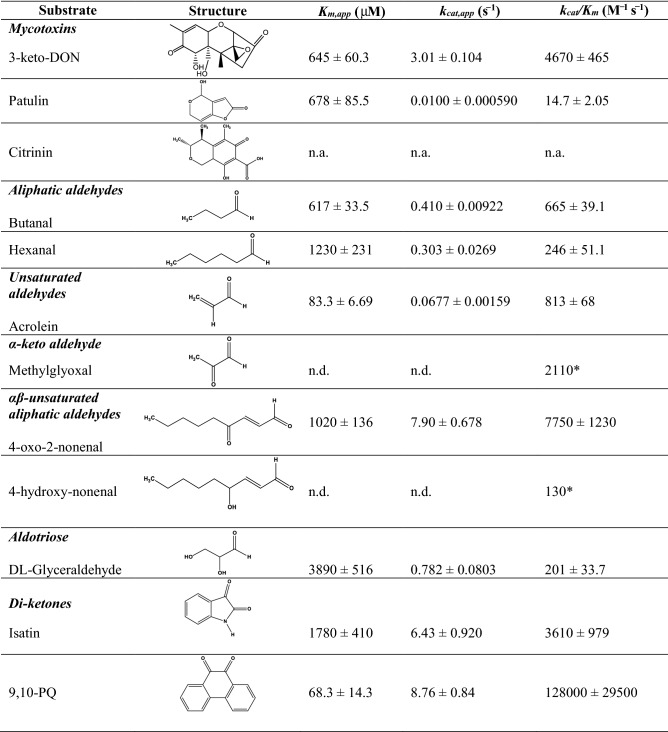
Substrate specificity profile of DepB_Rleg_.

Apparent kinetic parameters for reduction of model aldehydes and ketones by recombinant DepB_Rleg_ in the presence of 1 mM NADPH. (n.d. = not determined, n.a. = no activity, *estimated from the gradient of the v vs. [S] slope).

### Crystallization and structure determination of DepB_Rleg_

DepB_Rleg_ was successfully crystallized, and its structure was solved by molecular replacement using an AKR from *Polaromonas* sp. JS666 as a search model (PDB ID: 4XK2). Structural and refinement statistics are summarized in Table 2.

**Table 2:** X-ray diffraction data and refinement statistics.

**Data collection**	
Space group	*P*4 2_1_ 2
Cell dimensions	
* a, b, c* (Å)	159.45, 159.45, 163.90
α, β, γ (°)	90.00, 90.00, 90.00
Resolution (Å)	46.45–2.5 (2.589–2.5)*
Total reflections	1,202,979 (120,250)
No. of unique reflections	73,419 (7223)
*R*_merge_	0.2617 (2.503)
*I*/σ*I*	11.52 (1.30)
Completeness (%)	99.81 (99.81)
Redundancy	16.4 (16.6)
**Refinement**	
Resolution (Å)	
No. reflections	73,312 (7214)
*R*_work_/*R*_free_	0.1847/0.2341
No. atoms	2038
Protein	1275
Ligand/ion	188
Water	575
Wilson *B-*factors	47.35
*B*-factors	
Protein	61.26
Ligand/ion	89.53
Water	53.87
R.m.s deviations	
Bond lengths (Å)	0.002
Bond angles (°)	0.45
Ramachandran statistics	
Favored (%)	95.31
Allowed (%)	3.97
Outliers (%)	0.71

*Highest resolution shell is shown in parentheses.

**Figure 3:**
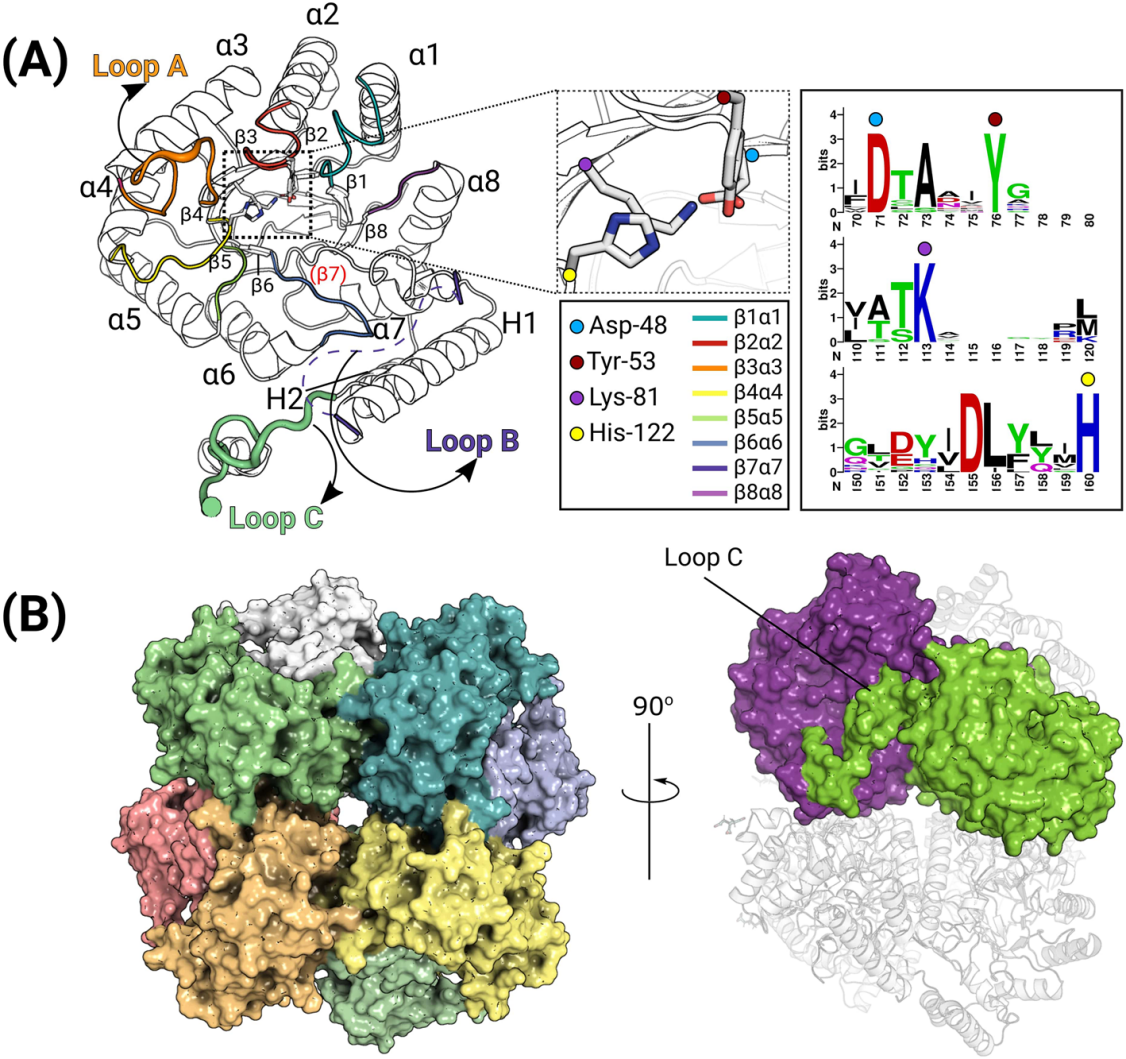
Crystal structure of DepB_Rleg_ protomer and octameric assembly. (**A**) Cartoon representation of the protomer with a view looking down the top of the TIM barrel rendered in black and white with the β7 annotated in red. Loop A (orange) caps the top of the substrate-binding cleft, loop B (dashes) could not be modeled but its position has been highlighted in this figure, and finally, loop C (green) which extends into the active site of the opposing protomer in the dimer. A sequence logo highlighting the conservation of the catalytic residues has also been provided. (**B**) Surface and cartoon rendition of the DepB_Rleg_ octamer. This assembly was predicted using the PISA server. View rotated at a 90° angle depicting 3D domain swapping of loop C at the dimer interface.

Four protomers of DepB_Rleg_ are present in the asymmetric unit designated as chains A, B, C, and D. Overall, the three-dimensional structure of each protomer adopts the classic triose-phosphate isomerase (TIM) barrel fold (α/β)_8_, except that the presence of Pro-204 in the region that would otherwise form strand β7 disrupts the hydrogen bonding pattern leaving this region as an extended loop. Two auxiliary helices designated as H1 and H2 are found at the periphery of the barrel, while the N-terminal end of the barrel is capped by an N-terminal β-hairpin loop. The TIM barrel scaffold contains βα loops that connect β-strands to α-helices and the αβ loops that connect α-helices to β-strands. βα loops are longer than αβ loops and impart structural and hence functional diversity to TIM barrel proteins such as AKRs^32^. The β3α3 loop (designated as Loop A) and the C-terminal loop (designated as Loop C) are proposed to govern substrate binding in AKRs while the primary role of the longer β7H1 loop (designated as Loop B) is for coenzyme binding^33^ (Fig. 3A). Due to a lack of clear electron density, the following residues within loop B could not be modeled: Chain A (214–241), Chain B (217–244), Chain C (219–245), and Chain D (218–245).

A pair of protomers (A and C, B and D) associate together forming a dimer with an average interface area of 2362.9 Å^2^, stabilized by 24 hydrogen bonds and 13 salt bridges. Dimerization involves 3D domain swapping with the exchange of the α-helix of loop C which interacts with loop A on the associating protomer. These dimers interact around the crystallographic four-fold symmetry axis to form a D_4_ symmetric octamer (Fig. 3B), with key interactions mediated by the N-terminal β-hairpin, α3, α4, and the β4α4 loop. PISA^34^ estimates that these interactions bury 748.6 Å^2^ and result in a solvation-free energy gain of -809.8 kcal/mol. The absolute molecular weight of DepB_Rleg_ was determined experimentally by SEC-MALS to be 325.6 ± 12.1 kDa which is consistent with the 317.5 kDa predicted for a DepB_Rleg_ octamer. (See Supplementary Fig. S3).

The closest structural homologs of DepB_Rleg_, are the functionally uncharacterized AKRs from the structural genomics initiative, *Polaromonas* sp. AKR (PDB ID: 4XK2, 44% sequence identity, 1.6 Å RMSD), an *Escherichia coli* AKR, Tas (PDB ID: 1LQA, 35% sequence identity, 2.0 Å RMSD), that has broad substrate specificity^35^, the beta subunit of the voltage-gated potassium channel from *Rattus norvegicus* (AKR6A2; PDB ID: 3EAU, 32% sequence identity, 2.1 Å RMSD), *E. coli* AKR14A1 (PDB ID: 4AUB, 35% sequence identity, 2.4 Å RMSD) that is specific towards methylglyoxal and diketones such as isatin^36^ and mithramycin side chain reductase from *Streptomyces argillaceus* (PDB ID: 6OW0, 33% sequence identity, 2.3 Å RMSD). Mithramycin side chain reductase is a ketoreductase that reduces the 4’ ketone side chain of mithramycin DK, an intermediate in the biosynthesis of the tricyclic antitumor polyketide, mithramycin^37^.

Diversity in certain loop regions was observed among these structural homologs. Differences in the conformation of loop B and the auxiliary helices, H1 and H2 could be due to conformational changes upon binding of NADP(H) and/or substrates. Loop B in the apo-structures of DepB_Rleg_ and *Polaromonas sp.* AKR is disordered, but clear electron densities for this region were observed for the NADP^+^ binary complexes of the other structural homologs. This is in agreement with the finding that loop B becomes ordered upon coenzyme binding^38^. In addition to these conformational differences, variations in loop length are also evident, particularly for the *E. coli* Tas protein which possesses a long α4β4 loop (24 residues) compared with DepB_Rleg_ (8 residues). The length and structure of DepB_Rleg_ loop C (26 residues) also differed with AKR6A2 (6 residues), Tas (5 residues), AKR14A1 (10 residues), and mithramycin side chain reductase (6 residues). In the hydroxysteroid reductase subfamily of AKRs truncation of this loop led to reduced specific activity towards steroid substrates, therefore the diversity in loop lengths among these AKRs likely contributes to corresponding differences in substrate specificity^39^.

### Coenzyme binding site

DepB_Rleg_ reduced 3-keto-DON using NADH and NADPH as coenzymes, although the catalytic efficiency with NADH was 40-times lower than with NADPH. The dissociation constant (*K*_*d*_) for NADH, as determined by tryptophan fluorescence quenching experiments, was about sixfold higher than NADPH. In contrast, most AKRs are NADPH specific with NADPH dissociation constants on average, 1000-fold lower than NADH^40^. DepB_Rleg_, therefore, shares the rare dual coenzyme specificity with a small number of enzymes from different AKR families, including the hydroxysteroid dehydrogenases (AKR1C)^41,42^, xylose reductases (AKR2B)^43–45^, beta subunit of voltage-gated potassium ion channels (AKR6A)^46^, aflatoxin reductases (AKR7A)^47^, and pyridoxal dehydrogenases (AKR15A)^48^. The structural basis for NADH specificity was studied for *Candida tenuis* xylose reductase (AKR2B5), revealing a key Glu-227 residue on loop B which forms bidentate hydrogen bonds with the 2’ and 3’ hydroxyl groups of the adenosine moiety of NAD^+^^44^. In DepB_Rleg,_ this residue is also conserved and corresponds to Glu-222. In the NADP^+^ bound complex of AKR2B5, the 2’ monophosphate of NADP^+^ interacts with a positively charged Arg-280, the peptide backbone of Asn-276, and Ser-275. These residues are not strictly conserved in DepB_Rleg_ and are instead replaced with Gln-294, Arg-290, and Ala-288, underscoring the general variability in NADP^+^ interactions among the AKRs.

**Figure 4:**
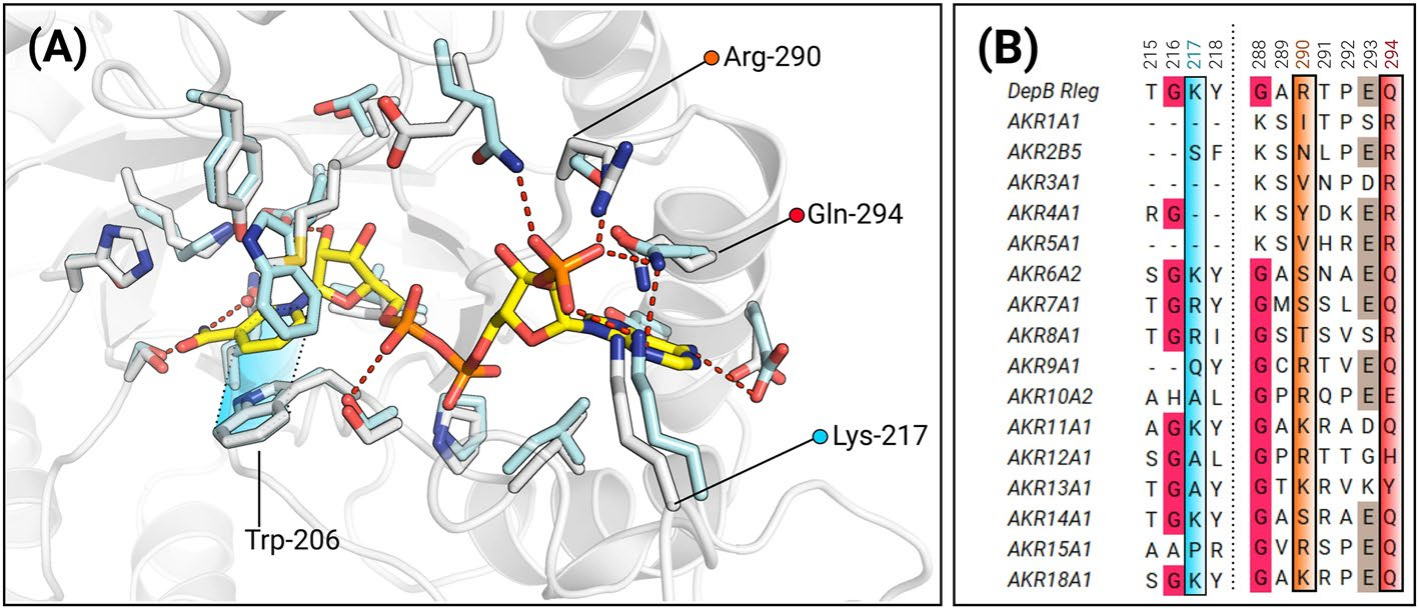
Model of the coenzyme binding site highlighting the conservation of putative coenzyme binding residues. (**A**) NADP^+^ was modeled by superimposing the ternary complex of AKR6A2 with apo-DepB_Rleg_. White sticks represent the residues from DepB_Rleg_ complex, while cyan sticks are residues corresponding to AKR6A2. Trp-206 has been re-oriented in the apo-DepB_Rleg_ structure to prevent a steric clash with the nicotinamide ring of NADP^+^. π-stacking interactions between the nicotinamide ring and Trp-206 are shaded in blue. In apo-DepB_Rleg_, Lys-217 points away from the entrance of the coenzyme binding pocket. However, by analogy to Lys-254 in AKR6A2, this residue may interact with at least one of the oxygen atoms of 2’monophosphate. (**B**) MSA depicting conservation of residues near the 2’monophosphate of the modeled NADP^+^ (See Supplementary Table S1 for accession codes).

Although NADP^+^ was present at 400 µM during the crystallization of DepB_Rleg,_ there was no electron density corresponding to this coenzyme. The putative coenzyme binding pocket was suitably defined and superimposed well with coenzyme binding sites of AKR binary complexes. AKRs bind NADPH in an extended *anti*-conformation to achieve the stereospecificity of hydride transfer^33^. An aromatic residue typically forms π-stacking interactions with the nicotinamide ring while the carboxamide moiety is oriented by highly conserved glutamine, serine, and asparagine residues. In DepB_Rleg_, these residues correspond to Trp-206, Gln-178, Ser-152, and Asn-153 respectively. In AKR6A2, the closest structural homolog of DepB_Rleg,_ the 2’monophosphate of NADP^+^ interacts with the side chains of Gln-62, Lys-254, Ser-325, and Gln-329, and (Fig. 4A). In DepB_Rleg_, these residues correspond to Glu-26, Lys-217, Arg-290, and Gln-294 respectively. To examine the conservation of these residues, a multiple sequence alignment was constructed with AKR family representatives. Lys-217 which is present on loop B is not strictly conserved and is often substituted for a basic residue or a smaller hydrophobic residue. Arg-290 is partially conserved across the AKR families and this position is often frequented by a basic residue or a polar hydrophilic residue. Gln-294 is strictly conserved across AKR6A2, AKR7A1, AKR9A1, AKR11A1, AKR14A1, AKR15A1, and AKR18A1 but for the other families, it is replaced with either a basic or aromatic residue (Fig. 4B). Lastly, Glu-26 was the least conserved and was therefore not selected for further analysis.

All recombinant N-terminal His-tagged DepB_Rleg_ coenzyme variants were purified by Ni-NTA chromatography with expected molecular weights of 39 kDa (See Supplementary Fig. S1). Replacements of Lys-217, Arg-290, and Gln-294 for negatively charged glutamate significantly altered the *K*_*d*_ for NADPH relative to wild type DepB_Rleg_, with no significant effect on the *K*_*d*_ of NADH. Among these variants, the R290E variant displayed an 11-fold increase, followed closely by Q294E with a ninefold increase and finally K217E with only a sixfold change (Table 3). Substitutions for neutral residues as in the case for R290N displayed a moderate change in the *K*_*d*_ by fivefold, while for K217M, the *K*_*d*_ change relative to the wild type was more pronounced with a nearly sevenfold change. Overall, for all the mutants, there was no appreciable change in *K*_*d*_ for NADH.

**Table 3:** Apparent coenzyme dissociation constants for purified recombinant DepB_Rleg_ and coenzyme variants.

Enzyme	*K*_*d*_ (µM)		*K*_*d*_^NADPH^/*K*_*d*_^NADH^
	NADPH	NADH	
DepB_Rleg_	11.4 ± 0.84	67.3 ± 2.5	0.17
R290E	123 ± 6.2	119 ± 9.8	1.03
R290N	59.5 ± 4.2	77.7 ± 3.6	0.76
Q294E	97.9 ± 13	91.2 ± 10	1.07
K217E	68.6 ± 8.7	87.1 ± 3.4	0.79
K217M	74.1 ± 4.6	82.9 ± 3.0	0.89

### Substrate-binding site

The catalytic mechanism of AKRs involves a stereospecific 4-*pro*-R hydride transfer from the nicotinamide ring of the coenzyme to the substrate carbonyl carbon, followed by protonation of the carbonyl oxygen by a tyrosyl residue^49^. This is facilitated by a proton relay with histidine or a lysine-aspartate pair^40^. In DepB_Rleg_, these catalytic residues are conserved: Asp-48, Tyr-53, Lys-81, and His-122. A water molecule occupies the position equivalent to the carbonyl oxygen group of the substrate and forms hydrogen bonds (2.6–2.9 Å) to the side chains of both His-122 and Tyr-53^49^. DepB_Rleg_’s substrate-binding pocket is lined with the following residues: Met-21, Asp-48, Val-52, Tyr-53, Lys-81, Arg-83, Phe-84, His-122, Ala-123, Ser-152, Asn-153 and Gln-180. Overall, DepB_Rleg_’s substrate-binding pocket is slightly larger and more hydrophobic in comparison with the NADP^+^- cortisone bound complex of AKR6A2. The physiological substrate of AKR6A2 is unknown, however, much like DepB_Rleg_ it also displays broad substrate specificity with aldehyde and ketone substrates^50^.

**Figure 5:**
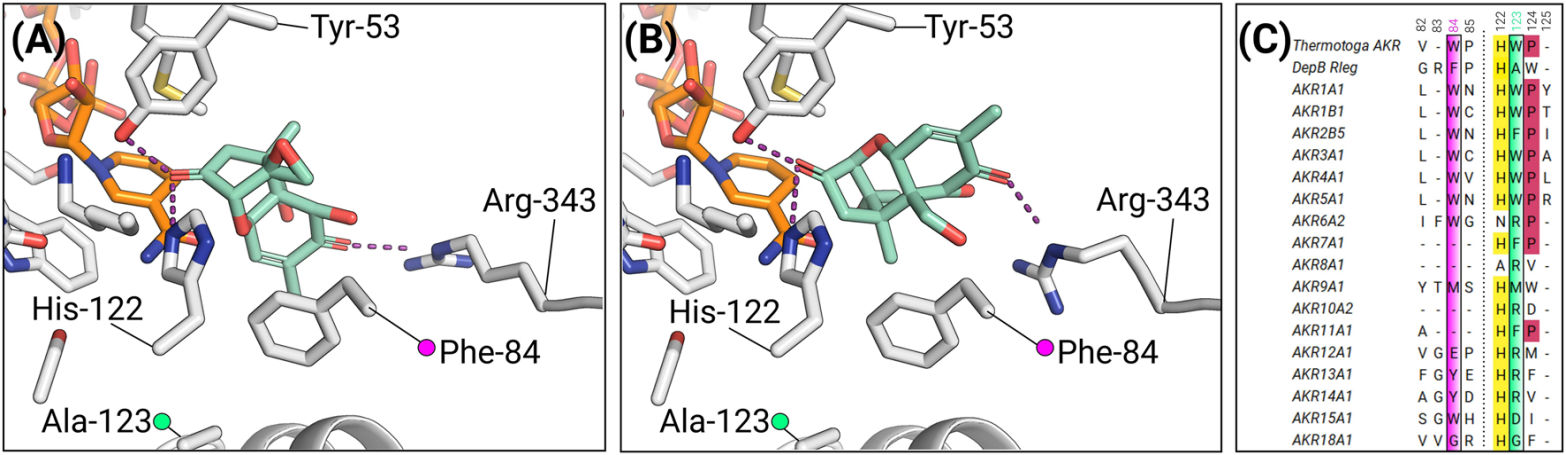
Model of the ternary complex of DepB_Rleg_. (**A**) Binding orientation for 3-keto-DON for hydride attack at the *re*-face. π-stacking interactions between Phe-84 and the cyclohexene ring are shaded yellow. (**B**) Binding orientation for 3-keto-DON for hydride attack at the *si*-face. (**C**) Multiple sequence alignments depicting the sequence variability at positions corresponding to Phe-84 and Ala-123 in DepB_Rleg_ with that of various AKR family representatives.

The stereospecificity of hydride transfer is strictly conserved in AKRs. In DepB_Rleg,_ the residues Ser-152, Asn-153, and Gln-178 are critical for maintaining the *anti*-conformation of the nicotinamide ring to achieve this stereospecificity^51^. However, hydride attack may occur either on the *re*-face or *si*-face of the prochiral carbonyl group. We determined by HPLC analysis that DepB_Rleg_ reduced 3-keto-DON to produce a diastereomeric ratio of 67.2% for 3-*epi*-DON and 32.8% for DON (See Supplementary Fig. S4 for HPLC chromatograms). To examine the molecular basis for this diastereoselectivity, we superimposed apo-DepB_Rleg_ with the recently solved *Debaryomyces nepalensis* xylose reductase (PDB ID: 5ZCM) complexed with a DTT-NADP^+^ adduct^52^. In this complex, the geometry of the C4N of NADP^+^ strongly resembles the puckered ring conformation of reduced NADPH. 3-keto-DON was then modeled into this complex in an orientation poised for hydride transfer to the *re*-face to produce 3-*epi*-DON. The carbonyl oxygen of 3-keto-DON was placed within hydrogen-bonding distance of Tyr-53 and His-122, while Arg-343 from Loop C was re-positioned to provide hydrogen bond contacts with the C8 ketone of 3-keto-DON. Phe-84, which is present on Loop A could potentially stack with the cyclohexene ring of 3-keto-DON (Fig. 5A). This residue corresponds to Trp-86 in *Thermotoga maritima* AKR, an enzyme that catalyzes the reduction of ethyl 2-oxo-4-phenylbutyrate to the *R*- and *S*-enantiomer of ethyl-2-hydroxy-4-phenylbutyrate^53^. In that study, the replacement of Trp-86 with smaller amino acids alleviates space constraints and enabled the enzyme to increase the production of the *R*-alcohol^53^. To produce DON, 3-keto-DON would require a 180° flip in the substrate-binding pocket (Fig. 5B). An observation gleaned from sequence analysis with other AKRs, is the presence of a bulky residue following the catalytic histidine (Fig. 5C). However, in DepB_Rleg_ this position is occupied by Ala-123 which eliminates any space constraints allowing for the accommodation of the C15 primary alcohol of 3-keto-DON. We note that in AKR18A1 which reduces 3-keto-DON to DON, a glycine residue occupies the positions corresponding to Phe-84 and Ala-123 providing a great deal of flexibility in its respective active site.

## Discussion

A comprehensive biochemical and structural analysis was conducted for DepB_Rleg_, a close homolog of DepB from *D. mutans* 17-2-E-8. This AKR catalyzes the critical step during DON detoxification to afford the significantly less toxic diastereomer, 3-*epi*-DON. SSN cluster analysis revealed that DepB_Rleg_ is a member of the recently discovered AKR18 family. Within this family, only preliminary biochemical characterization was conducted for AKR18A1 from *Sphingomonas* S3-4 suggesting its involvement in a DON detoxification pathway^22^. At a cutoff threshold of *e*^−57^, DepB_Rleg_ localizes to a functionally divergent cluster in the SSN comprised of AKR6, AKR12, and AKR14 families. AKR6 members comprise the cytosolic subunit of voltage-gated potassium ion channels and are proposed to modulate these channels via coenzyme binding^46^. AKR12 members are reductases involved in macrolide antibiotic synthesis^23–25^, while the AKR14A1 member identified in the SSN is proposed to be involved in methylglyoxal detoxification^54^. The gene organization as depicted in the GND indicates that *depB*_*Rleg*_ is not located in a similar biosynthetic operon. Instead, it is flanked by a putative MAO and several putative putrescine/spermidine transporter protein genes.

DepB_Rleg_’s substrate specificity was examined across a diverse range of model carbonyl substrates with its preferred coenzyme, NADPH. Amongst the ketone substrates, DepB_Rleg_ possesses the highest specificity constant for 9,10-PQ while comparable specificity constants were observed for the other diketone, isatin and 3-keto-DON. 9,10-PQ is a xenobiotic air pollutant that triggers the generation of reactive oxygen species (ROS) in vivo^12^ while isatin is an endogenous indole derived from tryptophan or phenylalanine metabolism by gut bacteria and has cytotoxic effects on microbes and various human cancer cell lines^55^. Overall, higher specificity constants were observed for ketones, versus aldehyde substrates. Among the lipid peroxidation substrates, DepB_Rleg_ displayed the highest specificity constant for 4-oxo-2-nonenal, followed by methylglyoxal and acrolein. DepB_Rleg_ had low specificity constants for 4-hydroxy-nonenal and DL-glyceraldehyde. The latter is typically a model substrate used for aldose reductases and aldehyde reductases from AKR1 families^17^. Surprisingly, DepB_Rleg_ can also reduce the mycotoxin patulin to E-ascladiol, an activity that was previously described only for the short-chain dehydrogenase family of oxidoreductases^56,57^. In summary, the broad substrate specificity of this AKR, therefore, lends credence to its secondary role in general detoxification processes.

A consensus among most AKRs is their preference for the coenzyme, NADPH over NADH^49^. Investigations of the coenzyme binding affinities of DepB_Rleg_ revealed that it can utilize both NADPH and NADH, albeit with preference for NADPH over NADH. Given that NADPH is more expensive than NADH, the ability of DepB_Rleg_ to utilize NADH as a coenzyme will be beneficial for its use as a biocatalyst to detoxify DON. Based on comparative structural analysis, the residues Lys-217, Arg-290, and Gln-294 are proposed as key contributing elements for DepB_Rleg_’s preference for NADPH. These structural elements likely contribute to a hydrogen bond network encompassing the 2’monophosphate of NADPH. Precedents for coenzyme specificity are well established for short-chain dehydrogenases (SDRs). In NADP^+^ dependent SDRs, a basic residue in the glycine-rich motif of the Rossman fold lies governs NADP^+^ specificity while for NAD^+^ dependent SDRs, typically an aspartate or glutamate is present^58^. Similarly, in DepB_Rleg_, the basic residues, Arg-290 and Lys-217 contribute the necessary salt-bridge interactions with the 2’monophosphate, while Gln-294 also provides hydrogen bond interactions and provides planar stacking interactions between the amide group of its side chain and the adenosine ring. Not surprisingly, substitutions for negatively charged residues generated electrostatic repulsion with the 2’monophosphate group resulting in an increased NADPH *K*_*d*_ value for R290E, Q294E, and K217E. While the change in *K*_*d*_ for the R290N variant was not as dramatic, we rationalize that replacement of the arginine for a polar, shorter residue like asparagine likely altered the hydrogen bond network coordinating the 2’monophosphate which consequently affected the binding mode of NADPH. K217M and K217E displayed a similar increase in *K*_*d*_ for NADPH, however, the effect was not as pronounced as in the Arg-290 variants. As Lys-217 is present on Loop B, which displays a great deal of sequence variance, it is plausible that a proximal, basic residue may have compensated for the absence of the lysine residue in the K217M and K217E variants.

Reduction of 3-keto-DON by DepB_Rleg_ produced 3-*epi*-DON and DON in diastereomeric ratios of 67.2% and 32.8% respectively. Modeling suggests that the formation of the two diastereomers is due to alternate 3-keto-DON binding modes that enable the *re* or *si*-face to approach the 4-*pro*-R hydrogen of the nicotinamide cofactor. Although DepB_Rleg_ does not show strict diastereoselectivity towards 3-*epi*-DON formation, coupling the enzyme with DepA which has an apparent *K*_*m*_ of 32 ± 4 µM for DON^9^, would drive the equilibrium towards 3-*epi*-DON formation.

In conclusion, this comprehensive analysis of DepB_Rleg_ provides extensive insights into the AKR18 family. As evidenced by this structure–function study, DepB_Rleg_ presents as a versatile biocatalyst owing to its broad substrate specificity and a large active site which can accommodate a range of toxic endogenous aldehydes and ketones. The availability of the crystal structure of the enzyme should also facilitate future protein engineering work in targeting residues in the vicinity of the proposed substrate-binding site to improve the diastereoselectivity of the enzyme. While the feasibility of employing DepB in DON detoxification applications remains a contentious issue due to its NADPH dependence, this study provides a translational framework to modify its coenzyme specificity.

## Materials and methods

### Chemicals

3-keto-DON, DON, and E-ascladiol were purchased from TripleBond (ON, Canada). Patulin, citrinin, 4-oxo-2-nonenal, and 4-hydroxynonenal were purchased from Cayman Chemicals (MI, USA). 3-epi-DON was previously purified and the identity was confirmed via LC-MS^6^. All other chemicals unless otherwise stated were from Sigma-Aldrich and Thermofisher.

### Protein expression and purification

His-tagged DepB_Rleg_ and variants were recombinantly expressed in *E.coli* BL21 LOBSTR (low background strain). This engineered strain minimizes background contamination by histidine-rich *E. coli* proteins such as SlyD and ArnA during Ni-NTA chromatography^59^. An overnight starter culture was used to inoculate 4L of LB media and cultures were grown at 37 °C with shaking speed at 200 RPM. Recombinant protein expression was induced with 1 mM isopropyl β-D-1-thiogalactopyranoside (IPTG) and incubated at 15 °C overnight with shaking. Cells were harvested by centrifugation and washed with 20 mM HEPES pH 8.0. The pellet was resuspended in 20 mM HEPES pH 8.0 buffer containing up to 1 mg/mL DNase I and lysed by 7–8 passages through a French press at 15,000 lb/in^2^. Cell debris was removed through centrifugation at 4 °C and the clarified lysate filtered through a 0.45 µm filter before incubation for 1 h at 4 °C with Ni^2+^-NTA resin in buffer (50 mM sodium phosphate buffer pH 8.0, 300 mM NaCl) with binding buffer (20 mM imidazole pH 8, 150 mM NaCl). The mixture was loaded onto a gravity column and washed with the same binding buffer. His-tagged proteins were eluted with 150 mM imidazole pH 8. Buffer exchange with 20 mM HEPES pH 7.5 with 10% glycerol and 150 mM NaCl was conducted in a stirred cell equipped with a YM10 filter (Amicon). Protein concentration was determined using a Bradford Assay^60^ with Bovine Serum Albumin used as a standard. The purity of the recombinant enzyme and the molecular weight of recombinant DepB_Rleg_ and coenzyme variants was estimated using Coomassie blue-stained SDS-PAGE.

### Molecular weight determination

The absolute molecular weight of DepB_Rleg_ was determined using Size Exclusion Chromatography coupled with Multi-Angle Light Scattering (SEC-MALS). The column utilized for size exclusion was a P3000 single-pore GPC/SEC column (Malvern Panalytical). The system was equilibrated with the following buffer system: 20 mM HEPES, 150 mM NaCl, 0.02% NaN_3_ at pH 7. The concentration of the protein samples was 0.5 mg/mL and samples were filtered through 0.2 µm filters prior to injection.

### Crystallization and X-ray diffraction collection

Crystallization conditions for DepB_Rleg_ were screened using the JCSG-plus™ kit (Molecular Dimensions) and an initial hit was obtained in condition B1 (0.1 M citrate pH 4, 0.8 M ammonium sulfate). Condition B1 was optimized and a crystal was obtained in the following optimized condition: 0.8 M ammonium sulfate, 20 mM ammonium acetate, and 0.1 M TRIS citrate. The protein was crystallized at 11 mg/mL in the presence of 400 µM NADP^+^ via sitting drop vapor diffusion at 288 K. X-ray diffraction data were collected at the Canadian Macromolecular Crystallography Facility (CMCF-BM), processed using XDS, and scaled using XSCALE^61^. Crystals diffracted to 2.5 Å, and belonged to the space group *P*42_1_2, with unit-cell parameters *a* = *b* = 159.45, *c* = 163.9 Å. The crystal structure of DepB_Rleg_ was solved using the automated molecular replacement pipeline, MrBUMP which generates an exhaustive list of search models. First, multiple sequence alignments are conducted between the query and homologous protein structures, second, the top hits are subjected to model preparation using a suite of CCP4 programs and finally, the models are submitted to Phaser for molecular replacement^62^. The best search model prepared from this process is the AKR from *Polaromonas* sp. JS666. AutoBuild and subsequent refinements were conducted in PHENIX^63^ and final model building was conducted in COOT^64^.

### Molecular determinants of coenzyme specificity

Structural alignments were conducted via the DALI server^65^. The ternary complex of the AKR6A2 member, *Rattus norvegicus* voltage-dependent K^+^ channel beta subunit (Kvβ) (PDB ID: 3EAU) was superimposed on to the apo-DepB_Rleg_ structure. Structures were visualized in PyMOL (Schrödinger, Inc.) and torsion angles of residues were adjusted in the DepB_Rleg_ structure to prevent steric clash with the 2’ monophosphate and the nicotinamide ring of NADP^+^. Putative residues involved in NADPH specificity were selected within a 4–5 Å distance from the 2’ monophosphate attached to the adenosine ribose. Multiple sequence alignments were constructed in UGENE^66^ using representative sequences from each AKR family and aligned using the MUSCLE algorithm^67^ Based on this analysis, the following residues were selected as candidates for mutagenesis: Lys-217, Arg-290 and Gln-294.

### Mutagenesis

Site-specific mutagenesis and mutagenic primer design were conducted as per the Single-Primer Reactions IN Parallel (SPRINP) protocol previously described^68^. The wild type DepB_Rleg_ plasmid construct (pET28a*DepB*_*Rleg*_) was used as a template with single point mutation oligonucleotide primers (Integrated DNA Technologies) and Q5 High-Fidelity DNA Polymerase (New England Biolabs). All variant constructs were confirmed by a double restriction digest (NdeI/HindIII) followed by sequence analysis conducted at (AFL) Laboratory Services Division at the University of Guelph. All primers used for mutagenesis are listed in Supplementary Table S2.

### SSN construction and cluster analysis

The AKR SSN was constructed as previously described^69^. The master list of AKR sequences was downloaded from UniProt using the PFAM identifier (PF00248). In total, 318,574 sequences were retrieved, and the dataset was reduced by iteratively clustering the sequences using the H-CD-HIT server^70^. Sequences were then filtered based on an amino acid length cutoff between 320–350 to remove fragments and AKRs part of large multi-domain complexes. Finally, curated sequences from the AKR Database (https://hosting.med.upenn.edu/akr/) and the PDB were also added to the list of sequences, and any duplicates were removed by submission to CD-HIT with the sequence identity cut-off parameter set at 100%. An in-house BLAST search against this finalized database was then conducted at varying E-values and the resulting network was visualized using Cytoscape^71^. An optimal stringency threshold was selected to minimize the extensive fragmentation of the network.

### Differential scanning fluorimetry

The thermal stability of wild-type DepB_Rleg_ and variants were assessed using SYPRO Orange while fluorescence was monitored with a StepOnePlus Real-Time PCR system (Applied Biosystems, Foster City, U.S.A.). Experiments were carried out in triplicate in 100 mM HEPES pH 7.5 with a final protein concentration of 0.3 mg/mL and 1X stock of 5000X Sypro Orange as previously described^72^ (See Supplementary Table 3).

### Tryptophan quenching assays

Dissociation constants (*K*_*d*_) of DepB_Rleg_ and each coenzyme was determined using a tryptophan fluorescence-quenching assay with the PTI Fluorimeter (with FelixGX software). Excitation and emission wavelengths of 295 nm and 335 nm respectively were used while slit widths were fixed at 4 nm. Titrations were performed in 20 mM HEPES pH 7.5 with 120 μg of protein. The absorbance of only protein and buffer was measured before the assay to minimize any inner filter effects. All assays were conducted in triplicate. The change in fluorescence (ΔF) relative to the control (protein and buffer) was determined and then plotted against the concentration of each respective coenzyme. The data were fitted to the following equation^73^ by nonlinear regression using GraphPad Prism Version 8 software to determine the *K*_*d*_:

$$\Delta {\text{F}} = \frac{{\Delta {\text{F}}_{\max } + \left[ {\text{L}} \right]}}{{K_{d} + [{\text{L}}]}},\;\; \Delta {\text{F}} = {\text{F}}_{0} - {\text{F}}$$

F is the protein fluorescence intensity at varying concentrations of either NADPH or NADH represented by [L], and F_0_ is the fluorescence intensity of the protein in the absence of coenzyme.

### In vitro enzyme assays

For substrate specificity experiments, reactions were carried out in 1 mL reaction volumes consisting of 100 mM HEPES pH 7.5 buffer with the NADPH concentration fixed at 1 mM. The kinetic parameters for NADPH and NADH were determined with DL-glyceraldehyde concentration fixed at 10 mM. Substrate utilization was monitored by the decrease in absorbance of NADPH or NADH at 380 nm (ɛ = 1132 M^−1^ cm^−1^) using the Varian Cary 100 spectrophotometer at 298 K. The rates of NADPH oxidation in the absence of enzyme were subtracted from the measured rates. NADPH was maintained under saturating conditions, for this reason, reactions were monitored at 380 nm due to the high absorbance of NADPH at 340 nm. Reactions were conducted in triplicate and initiated upon the addition of the enzyme ranging between 6 and 787 µg. One unit of enzyme is defined as the amount of enzyme required to oxidize 1 µmol of NADPH per minute. Each assay was monitored for a time period of 10 min. Stock solutions of substrates were prepared in dimethyl sulfoxide (DMSO) and the amount of substrate added to the cuvette was adjusted to ensure that DMSO concentrations were about 10% of the reaction volume to minimize inhibition of enzyme activity (no more than 9% reduction in enzyme activity).

### HPLC analysis

Samples and standards were analyzed using an HPLC system (Agilent Technology 1200 Series, Palo Alto, CA, USA) equipped with a quaternary pump, an inline degasser, and a diode array detector set at 218 nm. A Phenomenex® 4 µ Jupiter Proteo 90A (250 × 4.6 mm) with a C18 guard column (Torrance, CA, USA) was used for the separation. DON, 3-keto-DON, and 3-*epi*-DON were eluted using a binary mobile phase set at a flow rate of 1.0 mL/min. The composition of the mobile phase was acetonitrile: water (10:90) for DON/3-*epi*-DON and acetonitrile:water (20:80) for 3-keto-DON. The injection volume was 10 μL.

### LC–MS/MS for patulin degradation products

The LC–MS/MS analysis was conducted using a Thermo® Scientific Q-Exactive™ Orbitrap mass spectrometer equipped with a Vanquish™ Flex Binary UPLC System (Waltham, MA, USA). Kinetex F5 100A column (150 × 4.6 mm, 2.6 μm) (Phenomenex) was used for separation. The binary mobile phase consisted of solvent A (99.9% H_2_O/0.1% formic acid, and solvent B (99.9% acetonitrile/0.1% formic acid). The chromatographic elution conditions were as follows: 0–16 min—5% B; 16–17 min—5–100% B; 17–21 min—100% B; 21–22 min—100–5% B; 22–28 min—5% B. The column compartment was kept at 22 °C; the flow rate was set at 0.3 mL/min; the injection volume was 10 μL; UV = 276 nm. The heated electrospray ionization (HESI) source was used in positive mode for the ionization of the target compounds; DDMS (top 10) mode was used, with NCE set at 30.

### Diastereoselectivity assays

DepB_Rleg_ at a final concentration of 9 µg/mL was incubated with 170 µM of 3-keto-DON, 400 µM NADPH and 50 mM TRIS pH 8, 150 mM NaCl. The reaction was incubated overnight and quenched with acetonitrile. Tubes were vortexed and left to stand for 10 min before filtration with a 0.45 µm filter. Samples were stored at − 20 °C until ready for HPLC analysis.

## Supplementary Information

Supplementary Information.

## Data Availability

Coordinates for the DepB_Rleg_ structure have been deposited in the Worldwide Protein Data Bank (wwPDB) with the PDB accession code: 7UTF. Data from this study can be requested from the corresponding author Stephen YK Seah.
