# Supplementary material for: Structure–function characterization of an aldo–keto reductase involved in detoxification of the mycotoxin, deoxynivalenol
Source: Sci Rep. 2022 Aug 30;12:14737. doi: 10.1038/s41598-022-19040-8 (PMC9427786; doi:10.1038/s41598-022-19040-8)
Supplement: Supplementary file 1 — Supplementary Information. [file 41598_2022_19040_MOESM1_ESM.docx]

**Structure-Function characterization of an aldo-keto reductase involved in detoxification of the mycotoxin, deoxynivalenol**

Nadine Abraham^1,2^, Kurt L. Schroeter^1^, Yan Zhu^2^, Jonathan Chan^1,2^, Natasha Evans^1,2^, Matthew S. Kimber^1^, Jason Carere^2^, Ting Zhou^2^ and Stephen YK Seah^1^*

^1^Department of Molecular and Cellular Biology, University of Guelph, Canada

^2^Guelph Research and Development Centre, Agriculture and Agri-Food Canada, Guelph, ON, Canada

*Dr. Stephen Seah, Email: sseah@uoguelph.ca; Tel: 1 (519) 824-4120 Ext: 56750

**Supplementary Figures**

**
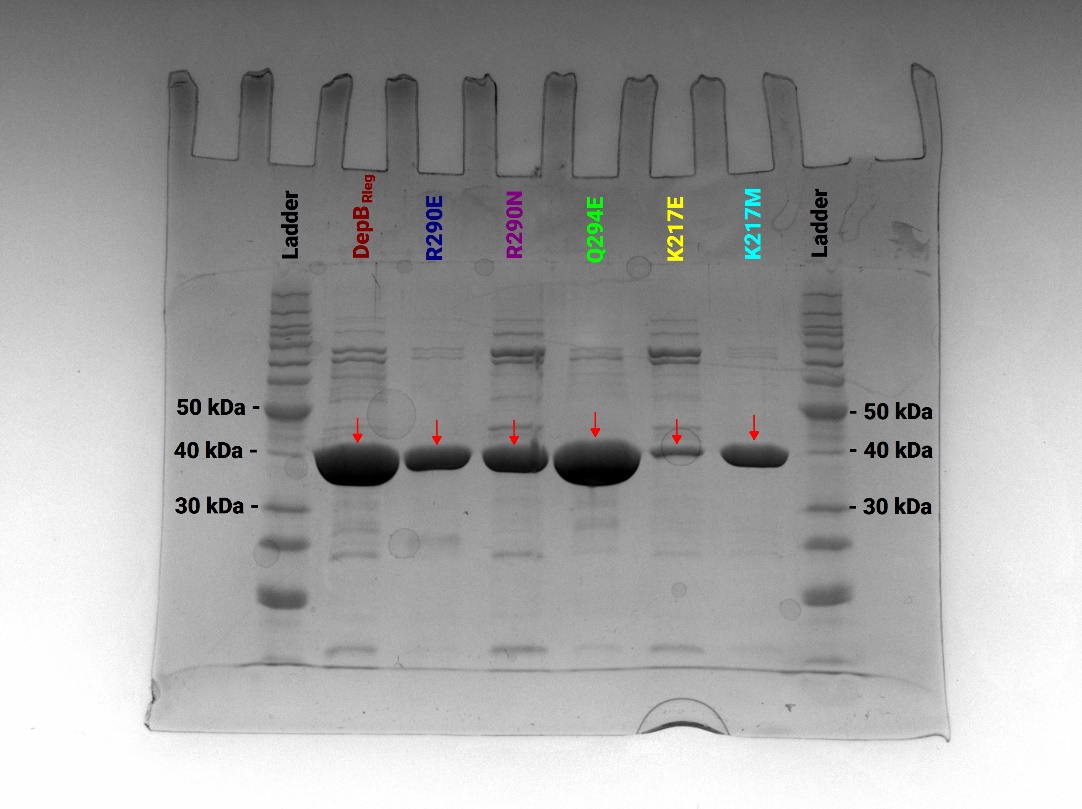
**

**Figure S1. A compilation of coomassie blue stained 10% SDS-PAGE gel of DepB_Rleg_ and coenzyme variants.** Benchmark^TM^ protein ladder was used as a molecular weight marker (MW). The remaining lanes are fractions for concentrated protein obtained from the 150 mM imidazole elution during Ni^2+^-NTA chromatography. All recombinant proteins were produced from *E.coli* BL21 LOBSTR.


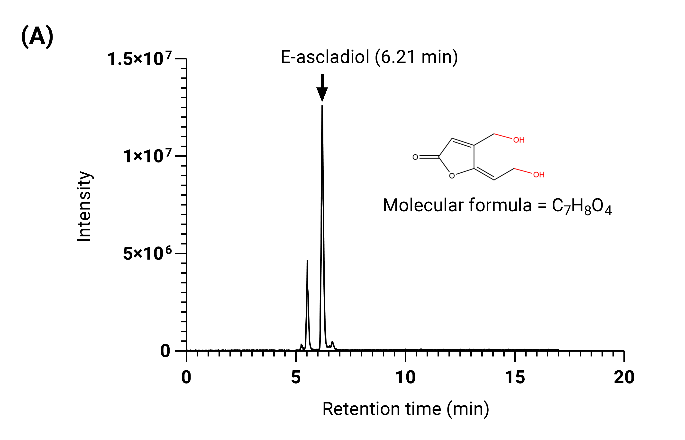

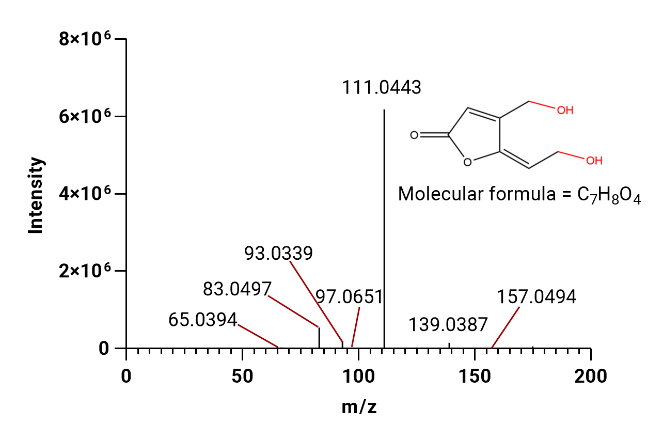


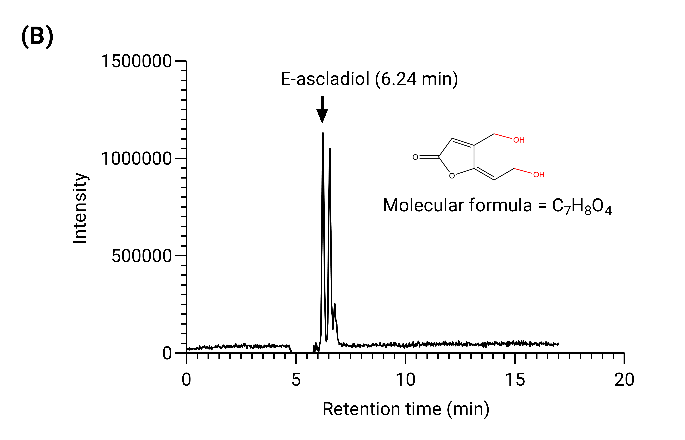

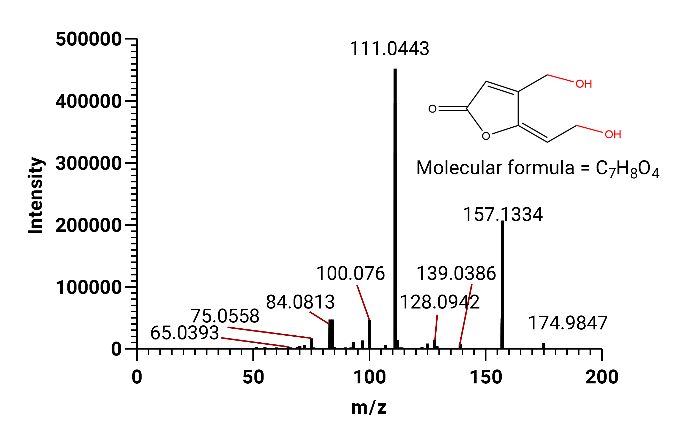


**Figure S2. LC-MS/MS results for E-ascladol formation by DepB_Rleg_.** (**A**) Extracted ion chromatogram (XIC) and MS/MS results for E-ascladiol standard. RT for E-ascladiol from the extracted ion chromatogram (XIC) is 6.21 min. ([M+H]^+^, C_7_H_8_O_4_, m/z = 157.0494) (**B**)**.** Extracted ion chromatogram (XIC) and MS/MS results for E-ascladiol formation (RT = 6.24 min) following an end-point assay with 0.73 mg/ml of DepB_Rleg_ in the presence of 500 µM NADPH and 10 ppm patulin. ([M+H]^+^, C_7_H_8_O_4_, m/z = 157.1334).


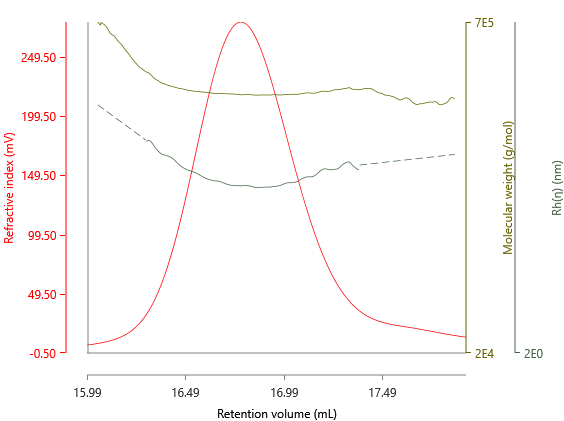


**3.98**

**325.6 ±
12.1 kDa**

**Figure S3. SEC-MALS of DepB_Rleg_.** The chromatogram depicts the molecular weight of DepB_Rleg_ (brown line) as well as the hydrodynamic radius (grey line). The OMNISEC Resolve (GPC/SEC system) and OMNISEC Reveal system (integrated multi-detector module) were utilized as per the manufacturer’s recommendations.


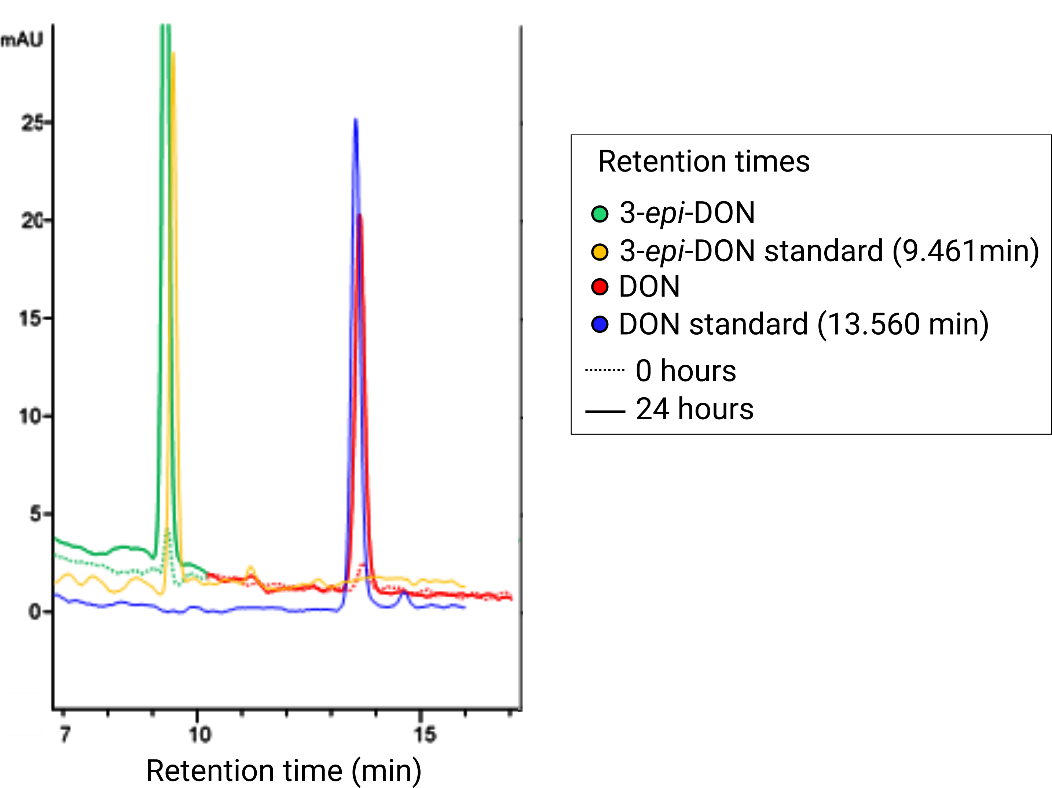


**Figure S4. HPLC chromatogram of DON and 3-*epi*-DON.** The chromatogram depicts the appearance of 3-*epi*-DON (RT = 9.293 minutes) and DON (Retention time = 13.642 minutes) after a 24-hour incubation with DepB_Rleg_. Retention times of the standards have also been provided.

**Supplementary Tables**

**Table S1. Accession IDs for AKR protein sequences utilized for multiple sequence alignments.** The following protein sequences were excluded for the generation of sequence logos: AKR6A2, AKR8A1. Sequences for AKR16 and AKR17 were not available for use in the multiple sequence alignment.

| AKR name | Protein name | Species | Accession number |
| --- | --- | --- | --- |
| 1A1 | Mammalian aldehyde reductase | *Homo sapiens* | NP_006057 |
| 2B5 | Xylose reductase | *Candida tenuis* | AAC25601 |
| 3A1 | Gcy1p | *Saccharomyces cerevisiae* | P14065 |
| 4A1 | 6'-deoxychalcone synthase | *Glycine max* | P26690 |
| 5A1 | Reductase | *Leishmania major* | P22045 |
| 6A2 | Shaker channel β-subunit (Kvb2) | *Rattus norvegicus* | CAA54142 |
| 7A1 | Rat liver aflatoxin aldehyde reductase | *Rattus norvegicus* | P38918 |
| 8A1 | Pyridoxal reductase | *Schizosaccharomyces pombe* | O14295 |
| 9A1 | Sterogmatocystin dehydrogenase (StcV) | *Aspergillus nidulans* | Q00727 |
| 10A2 | Streptomycin AKR (StrT) | *Streptomyces glaucescens* | CAA07384 |
| 11A1 | Vegetative protein 147 | *Bacillus subtilis* | P46336 |
| 12A1 | NDP-hexose-2,3-enoyl-reductase TylCII | *Streptomyces fradiae* | AAD41821 |
| 13A1 | YakC AKR | *Schizosaccharomyces pombe* | Q09923 |
| 14A1 | Aldehyde reductase | *Escherichia coli* | AAA69168 |
| 15A1 | Pyridoxal dehydrogenase | *Microbacteriun luteolum* | BAC97800 |
| 18A1 | Putative aryl alcohol dehydrogenase in DON detoxification | *Sphingomonas* sp. S3-4 | ASY03293.1 |
| Not classified | *Thermotoga maritima* AKR | *Thermotoga maritima* | Q9X265 |
| 18A2 | DepB_Rleg_ | *Rhizobium leguminosarum* | J0WHR2 |

**Table S2.** Primers used for site specific mutagenesis.

| Variant | Oligonucleotide sequence |
| --- | --- |
| R290EF | 5’-GTTATTCTGGGTGCG**GAG**ACCCCGGAACAGCTG-3’ |
| R290ER | 5’-CAGCTGTTCCGGGGT**CTC**CGCACCCAGAATAAC-3’ |
| R290NF | 5’-CTGGGTGCG**AA**TACCCCGGAACAGCTGG-3’ |
| R290NR | 5’-CCGGGGTA**TT**CGCACCCAGAATAACGCTGGTGATCG-3’ |
| Q294EF | 5’-ACCCCGGAA**G**AGCTGGCGGACAACCTGGGT-3’ |
| Q294ER | 5’-CGCCAGCT**C**TTCCGGGGTACGCGCACCCA-3’ |
| K217EF | 5’-CTGACCGGC**G**AGTACAAACGTGATGAAATG-3’ |
| K217ER | 5’-GTTTGTACT**C**GCCGGTCAGCCAACCG-3’ |
| K217MF | 5’-GGTTGGCTGACCGGCA**T**GTACAAACGTGAT-3’ |
| K217MR | 5’-ATCACGTTTGTAC**A**TGCCGGTCAGCCAACC-3’ |

**Table S3.** Melting temperatures for wild type DepB_Rleg_ and coenzyme variants.

| **Enzyme** | **T_m_ (^o^C)** | **ΔT_m_** |
| --- | --- | --- |
| DepB_Rleg_ | 51.7± 0.00757 | 0 |
| R290E | 51.7± 0.00757 | 0 |
| R290N | 44.7± 0.00683 | 7 |
| Q294E | 47.8 ± 0.00220 | 3.9 |
| K217E | 41.7 ± 0.00510 | 10 |
| K217M | 44.7 ± 0.00683 | 7 |
